# Femtosecond laser-assisted cataract surgery: Update and perspectives

**DOI:** 10.3389/fmed.2023.1131314

**Published:** 2023-03-02

**Authors:** Mateusz Kecik, Cedric Schweitzer

**Affiliations:** ^1^Department of Ophthalmology, Hopitaux Universitaires de Genève (HUG), Genève, Switzerland; ^2^Department of Ophthalmology, CHU Bordeaux, Bordeaux, France; ^3^INSERM, Bordeaux Population Health Research Center, Team LEHA, UMR 1219, Univ. Bordeaux, Bordeaux, France

**Keywords:** cataract, cataract surgery, femtosecond laser (fs), femtosecond laser assisted cataract surgery (FLACS), phacoemulsification

## Abstract

Cataract surgery is among the most frequently performed surgical procedures worldwide and has a tremendous impact on patients' quality of life. Phacoemulsification (PCS) is accepted as a standard of care; its technique has continuously evolved and already achieved good anatomical, visual, and refractive outcomes. Lasers in ophthalmology are widely used in clinical practice, femtosecond lasers (FSLs) for corneal surgery in particular. It was natural to assess the usefulness of FSL in cataract surgery as this technology was within reach. Indeed, precise and reproducible cuttings provided by FSL platforms could improve standardization of care and limit the risk associated with the human element in surgery and provide a step toward robot-assisted surgery. After docking and planning the procedure, femtosecond lasers are used to perform corneal incisions, capsulorhexis, lens fragmentation, and arcuate incisions in an automated manner. A well-constructed corneal incision is primordial as it offers safety during the procedure, self-seals afterward, and influences the refractive outcome. Capsulorhexis size, centration, and resistance to shearing influence the surgery, intraocular lens (IOL) centration and stability, and posterior capsular opacification formation. Lens fragmentation is where most of the energy is delivered into the eye, and its amount influences endothelial cell damage and potential damage to other ocular structures. The arcuate incisions offer an additional opportunity to influence postoperative astigmatism. Femtosecond laser-assisted cataract surgery (FLACS) has been a topic of research in many studies and clinical trials that attempted to assess its potential benefits and cost-effectiveness over PCS and is the subject of this mini-review.

## Introduction

Cataract remains the leading cause of blindness and the second cause of moderate and severe visual impairment in 2020, affecting 15.2 and 78.8 million people, respectively, worldwide. Cataract surgery is the most routinely performed surgical procedure, with 7 million surgeries performed per year in Europe, 3.7 million in the United States, and 20 million worldwide (1). Since its first introduction in 1967 by Kelman and continuous development of machines and intraocular lenses, phacoemulsification cataract surgery (PCS) has become the standard of care (2–6).

The recent development of near-infrared lasers with ultrashort pulse duration of the femtosecond domain (10^−15^ s) has opened new insights in ophthalmic surgery. Femtosecond lasers (FSLs) had their first clinical application in ophthalmology in corneal treatment in the late 1990s; they were then evaluated for their role in cataract surgery (7), with first treated patients in 2009 (8) and received Food and Drug Administration approval in 2010. During FLACS, after the docking procedure and owing to non-invasive and precise tissue treatment (9), FSL can be used to perform corneal incisions, capsulotomy, lens fragmentation, and arcuate incisions. Eliminating the human element from the first steps of the surgery has been suggested to improve the safety and reproducibility of surgical outcomes (10, 11). However, well-conducted and sufficiently powered randomized clinical trials comparing both techniques were needed to provide robust evidence and recommendations (12).

In this article, we report on the principles of FLACS and review current scientific evidence on PCS and its potential perspectives.

## Technical performances and principles of FLACS

Femtosecond laser is a near-infrared (1,053 nm) solid-state laser achieving photodisruption in ocular tissues; it is capable of delivering ultrashort femtosecond pulses 200–800 fs (1 fs = 10^−15^ s) of low overall energy and high peak power. It leads to more precise impact, less energy per pulse, and, in turn, less damage in collateral tissue than longer pulse lasers, e.g., nanosecond lasers (13). To achieve photodisruption, a certain degree of energy threshold must be surpassed, depending on the nature of treated tissue and different laser features: numerical aperture (NA) and wavelength. A larger NA decreases the volume of the focal spot and, together with a shorter wavelength (e.g., in the UV range for new prototype femtosecond lasers), lowers the energy threshold. A shorter pulse laser enables a decrease in the total energy needed to achieve this threshold. Photodisruption consists of three consecutive events: plasma formation, shock wave generation, and cavitation. Rapidly expanding plasma leads to the creation of a focal shock wave, which is atraumatic for the surrounding tissue and leaves behind only a small residual gas bubble (14). The mechanism of tissue separation achieved by FSL is twofold: ablation and cleaving. Gas bubbles represent directly ablated tissue by plasma formation within the laser focus, while rapid expansion and cavitation achieve further tissue separation by cleaving. Laser parameters determine which of those two mechanisms plays a chief role in tissue separation. At higher pulse energies, the radius of disrupted tissue is greater than the spot size, and tissue is separated by mechanical forces of expanding gas bubbles. Lower energies generate less cavitation and ablate a smaller volume of tissue; they allow for less traumatic tissue separation but require much tighter spot placement and call for higher pulse frequencies.

The cataract laser faces challenges different from the corneal platforms. Treatment depths in the former are usually between 100 and 130 μm, as with LASIK, while cataract surgery demands flexibility up to 7,500 μm. Such a difference generates the need for increased spot size and higher pulse energy with the cataract lasers. In addition, 5–10 times greater total energy and spot placements are required for lens surgery, in comparison to corneal surgery (8).

In FLACS, complicated and flexible laser patterns are needed, as each step of cataract surgery requires its proprietary treatment and energy profile. Laser capsulotomy requires an adjustable circular laser incision (achieved with postage stamp perforations) at a considerable depth with good accuracy. Lens fragmentation enables the surgeon to choose between different patterns of lens softening: cubes, spheres, pies, etc. So far, no ideal pattern enabling a low cumulative dissipated energy (CDE) has been described, but some lens softening patterns may be better than others in different grades or types of cataract (15).

The patient interface (PI) is essential to connect the laser to the eye and therefore must provide adequate stability without distortion of eye structures, be well tolerated by the patient, and not interfere with laser treatment. In FLACS, either a curved lens applanation or a fluid interface is used. Studies comparing docking with a curved vs. fluid PI showed no significant differences in capsulotomy regularity (16), but corneal folds, sometimes occurring with applanating PI, may induce incomplete capsulotomies with subsequent anterior capsular tears (17). The non-applanating designs, using a suction ring attached to the sclera and a fluid immersion chamber, do require more corneal exposure while docking but offer a larger treatment area, better compensation for lens tilt, and a much lower induced IOP rise during the treatment (17). Speculation exists whether the anterior movement of the lens, anterior hyaloid, and vitreous base during suction can predispose patients to develop vitreoretinal pathology, but no evidence from corneal-only FSL exists (18). The docking procedure is paramount in achieving good laser treatment but is an extra step compared to PCS and can be time-consuming (19, 20). A potential complication is the loss of suction leading to incomplete laser treatment. In an early study by Bali et al. (20), the authors experienced suction loss requiring abandoning the procedure in 2.5% of cases, but the study demonstrated a learning curve for the first 100 cases. Manning et al. (21) indicated that the laser procedure had to be abandoned in 0.1% (3 eyes). Zhang et al. (22) found the completion of capsulotomy, lens fragmentation, and corneal incisions to be at 98.6, 98.5, and 97.6%, respectively. In the first studies, the most frequently reported complication related to docking was transient conjunctival redness or hemorrhage in up to 34% of cases (23).

Finally, unlike FSL corneal procedures, precise and reliable imaging is needed for FLACS procedures. OCT is the most frequent imaging technology, but three-dimensional confocal structured illumination imaging is also used by one platform. Indeed, by enabling a three-dimensional reconstruction of anterior eye structures, it helps define capsulotomy margins, corneal incision placement, and safety zones within the lens itself.

## Clinical results

### Corneal incisions

Incisions are a crucial step in completing cataract surgery with clear corneal incisions being the standard for PCS and FLACS. Laser-created incisions were found to be reproducible and stable and did not significantly change the high-order aberrations over time (24–27). Users can also skip this step and elect to perform them with the microkeratome; this seemed to be the common procedure among surgeons as indicated in the 2016 study from the European Registry of Quality Outcomes for Cataract and Refractive Surgery, since in only 34.7% of FLACS cases, the incisions were performed by laser (21). The reason might be due to the fact that laser incisions take significantly longer to perform. The laser-created incisions show a saw-tooth pattern, increased cell apoptosis, and are less smooth than manual incisions (28). They may be less stable in the early postoperative period as well (29).

Diakonis et al. (30) did not show any significant difference in surgically induced astigmatism between FLACS and PCS using a multivariate vectorial analysis.

### Capsulotomy

The main advantage of FLACS is the ability to create a perfectly circular, centered capsulotomy with an accurate and reproducible diameter (31–33). Anterior capsulotomy conditions both the remainder of cataract surgery and the refractive outcome achieved by patients. Inaccurate prediction of effective lens position (ELP), which is, in turn, affected by capsulotomy size and morphology, is one of the biggest factors leading to residual refractive error contributing to 35% of total residual error (34). Studies have confirmed femtosecond laser capsulotomies to be rounder, more precise, better centered, and more stable over time as compared to manual capsulorhexis. However, this advantage has not been translated into better visual acuity or effective lens position (35).

There are, however, disadvantages to a laser capsulotomy, mainly its strength. Electron microscopy images demonstrate that the laser impacts do not produce a smooth capsulotomy rim as in a manual rhexis but instead produces one with numerous notches that could be more prone to tearing (36), resulting in an overall lower tensile strength (37) and, in turn, a higher frequency of anterior capsular tears (up to 4% in early published studies) (23, 38). Completeness of anterior capsulotomy can also be impeded by eye tremor during laser delivery, laser misfires, or corneal opacities such as scars or radial keratotomies (38, 39), but the latter might be remedied by adjusting laser settings (39, 40). Laser settings seem to be key even in routine FLACS, as different lasers produce microscopically different capsulotomy edges which might explain the variability of capsulotomy completeness and anterior radial tears in literature (0.1–4%) (23, 31, 41).

Intraoperative miosis following docking and laser procedure has also been reported in one-third of cases (23). This is mainly related to prostaglandin release during laser treatment of the anterior capsule (42, 43). Factors associated with intraoperative miosis are older patient age, longer duration of laser treatment, smaller laser capsulotomy–pupil margin distance, shallower anterior chamber, and smaller preoperative pupil size (44, 45). Some preoperative treatment regimens have been proposed to reduce intraoperative miosis onset or severity: mainly topical non-steroidal anti-inflammatory drugs 1–4 days before surgery and the day of the surgery (46–48).

### Femtosecond laser-assisted arcuate keratotomy (FSAK)

Femtosecond laser-assisted cataract surgery has an advantage of an optional step of performing FSAK to correct low-to-moderate astigmatism during cataract surgery. FSAK has been found to be effective, safe, and stable over time (49–51). They also offer some flexibility as the surgeon can elect to open them at the slit-lamp during the postoperative period for a greater corrective effect.

Clinical studies have found FSAK to be comparable to toric IOLs in mild-to-moderate, central, and with-the-rule astigmatism correction but less predictable in moderate-to-high, against-the-rule, or limbus-to-limbus astigmatism (52, 53). A recent study by Hernandez et al. (54) found the toric IOL to be superior over FSAK in the correction of moderate (1.25–3.0 diopter) astigmatism. Nevertheless, FSAK is more predictable in comparison to manual limbal relaxing incisions, and the development of new nomograms in the future may improve their efficacy (49).

### Endothelial cell loss, ultrasound energy delivered in the eye, and difficult cases

A potential advantage of FLACS over PCS could be that the lens softening allows for the reduction in effective phacoemulsification time (EPT) and cumulative dissipated energy (CDE) and thus may result in less damage to ocular tissues, particularly the corneal endothelium. An early study by Abell et al. (55) demonstrated a 96.2% reduction in EPT in the optimized FLACS group associated with a 36.1% reduction in endothelial cell loss; the laser pretreatment enabled an ultrasound-free lens aspiration in 30% of patients.

On the contrary, the FEMCAT trial did not observe any difference in central corneal thickness or corneal endothelial cell count between FLACS and PCS (56), and the FACT trial even showed more cell loss in the FLACS group (57). Other clinical trials seem to agree with this observation (58, 59), and the 2022 report by the American Academy of Ophthalmology concluded that the adequately powered RTCs did not demonstrate a significant difference in endothelial cell loss between FLACS and PCS (60).

There might, however, exist groups of patients that could benefit from the decreased EPT more than those with a routine cataract. Patients with hard nuclear cataracts or with shallow anterior chambers were shown to achieve a faster visual recovery after FLACS (61, 62). Yong et al. (63) found a significant difference in endothelial cell loss among Fuchs' patients (mean of 15.3 ± 17.5% for PCS and 4.4 ± 25.0% for FLACS) that was even more significant in the mild cataract subgroup.

### Pediatric cataracts

Cataract surgery in infants differs from age-related phacosclerosis mainly because of the elasticity of the anterior capsule, the softness of the lens nucleus, and the necessity to perform posterior capsulotomy (64). FLACS has already been used in congenital cataract surgery with success (65–67), including challenging cases like Peters anomaly type 2 and persistent hyperplastic primary vitreous (68, 69). Femtosecond laser use is off-label in children and requires some adaptations in the surgical procedure. It creates capsulotomies that are larger than expected because of the tissue elasticity, and corrective factors must be used in treatment planning (66, 67). A laser-assisted posterior capsulotomy is feasible but requires redocking after lens aspiration (65).

### Cystoid macular edema and mediators of inflammation release

Cystoid macular edema (CME) is a relatively infrequent complication of any intraocular surgery with an incidence of 0.95% after cataract surgery (70). FLACS was seen as a potential way of limiting this complication by decreasing EPT. However, a release of prostaglandins into the aqueous humor has been demonstrated during FLACS, especially after capsulotomy (42, 43). This can result in intraoperative miosis increasing risks and surgical time during cataract removal and impacting CME formation through inflammation cascade and disruption of the blood–retina barrier (71, 72). In clinical studies, this relationship was not as straightforward, as Koo et al. (73) found FLACS to be associated with a lesser likelihood of the usage of a pupil expansion device in comparison with PCS (7.0 vs. 24.2%) and a faster CME resolution in FLACS in cases of pseudoexfoliation.

### Posterior capsule opacification (PCO) and phimosis

FLACS produces reliable capsulotomies contributing to bag stability and elimination of lens epithelial cells (LEC) and has been shown to induce LEC apoptosis in LEC close to the capsulotomy edge, which is linearly correlated with laser energy parameters and duration (74–76). This effect could theoretically decrease PCO rates in FLACS. Few results are reported, and some studies have even found higher PCO rates in FLACS compared to PCS and reported early optic axis opacities (21, 77), possibly in relation to the mesenchymal transformation of LEC when exposed to adjacent laser treatment.

### Results of randomized controlled trials: FLACS vs. PCS

Several well-designed and rigorously conducted randomized controlled trials (RCTs) comparing FLACS to PCS have been published in the literature (56–59, 62, 78–84); their details are shown in Table 1. FEMCAT, FACT, and St. Thomas (56, 57, 82–84) trials evaluated corrected, uncorrected distance vision acuities, and refractive outcomes and found no statistically significant differences between both groups. Since complications in cataract surgery occur rarely, no RCT has been powered sufficiently for a meaningful analysis, and the published trials have not found differences in complications of FLACS compared to PCS, with the exception of St. Thomas trial, which found a statistically higher rate of posterior capsule rupture in PCS (3 vs. 0%) (83). The endothelial cell loss was similar between FLACS and PCS in published RCTs.

**Table 1:** Summary of the main randomized clinical trials comparing phacoemulsification cataract surgery (PCS) and femtosecond laser-assisted cataract surgery (FLACS).

**Year published**	**Authors**	**Number of eyes included (completed follow-up)**		**Study design**	**Measured outcomes**	**Main significant findings**
		**FLACS**	**PCS**			
2013	Conrad-Hengerer et al. (78)	73	73	• 1 eye randomized to FLACS and the other to PCS	• ECL	• Lower endothelial cell loss in FLACS
				• Single surgeon	• CCT	• Reduction in corneal thickness with FLACS
				• 3-month follow-up		• Less EPT in FLACS
2014	Conrad-Hengerer et al. (79)	101	101	• 1 eye randomized to FLACS and the other to PCS	• CRT	• No difference in retinal thickness
				• Single surgeon	• Anterior chamber flare	• Less AC inflammation in FLACS immediately after surgery
				• 6-month follow-up		
2018	Bascaran et al. (58)	100 (92)	100 (92)	• 1 eye randomized to FLACS and the other to PCS	• ECL	• No differences in ECL, CCT, and endothelial cell features
				• Single surgeon	• CCT	• Less CDE and fluid requirements in FLACS
				• 6-month follow-up		• Eyes with complications excluded from the analysis
2018	Roberts et al. (82) St. Thomas Study	200 (198)	200 (198)	• 1 eye included per patient: randomized to PCS or FLACS	• Visual acuity (UCDVA, BCDVA, pinhole)	• No difference in visual acuity, refractive error, ECL, CCT, and CFT between the two groups
				• Single-center, 3 surgeons	• Refractive error	• More posterior capsule rupture in PCS (3 vs. 0%)
				• 3–4-week follow-up	• ECL, CCT, CRT	• 7 patients (3.5%) unable to receive FLACS
					• Complications	
2019	Krarup et al. (80)	96 (90)	96 (90)	• 1 eye randomized to FLACS and the other to PCS	• UCDVA, CDVA	• No difference in CDVA and UCDVA
				• Single surgeon	• ECL, hexagonality	• FLACS associated with a 21% lower ECL; no difference in hexagonality
				• 6-month follow-up		• Some patients with complications (nucleus drop, posterior capsule rupture, corneal edema) were excluded from the analysis
2019	Vasavada et al. (62)	91	91	• Eyes with shallow AC (< 2.5 mm); 1 eye randomized to FLACS and the other to PCS	• CCT	• Lower CCT in FLACS up to 1 month
				• Single center	• Corneal clarity	• No difference in ECD loss, CDVA, and UCDVA
				• 6 months follow-up	• ECL	• Less AC flare in the FLACS group
					• Anterior chamber flare	• Clearer corneas with FLACS on day 1
					• CDVA, UCDVA	
2020	Hansen et al. (81)	64	71	• Patients randomized to PCS or FLACS (unilateral or bilateral)	• BCVA	• No difference in BCVA, UCDVA, and ECL
				• Single center, surgery performed by resident surgeons (16)	• ECL	• Patient satisfaction, total intraocular operating time, and complications not significantly different between two groups
				• 3-month follow-up	• Complications	
					• Patient-reported outcomes	
					• CDE, fluid volume	
2020	Dzhaber et al. (59)	67	67	• 1 eye randomized to FLACS and the other to PCS	• ECL	• No difference in ECL and CCT
				• Single surgeon	• CCT	• Longer operating time, more Balanced Salt Solution use, longer cortex removal in FLACS
				• 3-month follow-up		
2020	Stanojcic et al. (83) St. Thomas Study	116	118	• Continuation of the St. Thomas Study	• Visual acuity (UCDVA, CDVA)	• No difference in visual acuity, refractive error, ECL and CCT
				• 12-month follow-up	• Refractive error	• No difference in posterior capsule opacification
					• ECL, CCT	• More effective astigmatic correction with intrastromal laser incisions compared to manual relaxing incisions
					• Complications	
2020	Schweitzer et al. (56) FEMCAT trial	704	685	• Patients randomized to receive FLACS or PCS (unilateral or bilateral)	• Composite outcome measure including:	• No difference in the overall success rate of the composite outcome/ No difference in BCVA, refractive error, corneal astigmatism changes, and complications
				• Multicenter (5) study in France; 21 surgeons	• BCVA	• No difference in ECL and CCT
				• 12-month follow-up	• Refractive error (absolute refractive error)	• FLACS not cost-effective: €10,703 saved per additional patient treated successfully with PCS
					• Change to magnitude and axis of corneal astigmatism	No significant difference in ECL, CMT, and anterior chamber flare
					• Complications	
					• Incremental cost-effectiveness ratio	
					• ECL, CMT, and anterior chamber flare	
2020	Day et al. (84) FACT trial	392 (353)	393 (317)	• Patients randomized to receive FLACS or PCS (unilateral or bilateral)	• UCDVA, CDVA	• Similar visual acuity, refractive error, and patient satisfaction outcomes
				• Multicenter (3) study in the UK	• Complications	• Similar rate of intraoperative complications
				• 3-month follow-up	• Patient-reported outcomes	• Higher ECL in the FLACS group that nearly reached statistical significance (p= 0.06)
2020	Day et al. (57) FACT trial	392 (311)	393 (292)	• Continuation of FACT trial	• UCDVA, CDVA	• Similar visual acuity and patient satisfaction results
				• 12-month follow-up	• ECL	• No difference in complication rates
					• Complications	• No difference in ECL
					• Patient-reported outcomes	• FLACS was not cost-effective

AC, anterior chamber; BCVA, best-corrected distance visual acuity; CCT, central corneal thickness; CDE, cumulative dissipated energy; CDVA, corrected distance visual acuity; CRT, central retinal thickness; ECL, endothelial cell loss; EPT, effective phacoemulsification time; UCVA, uncorrected distance visual acuity.

### Cost-effectiveness

The FLACS technology is expensive both for the hospital in relation to the initial cost of the laser, space, and personnel and for the patient due to the cost of consumables (85). In addition, FLACS still requires the use of a phacoemulsification-aspiration machine to remove lens pieces or viscoelastic devices. Cost-effectiveness is a key aspect of integrating a surgical procedure in the medical landscape; only a few studies examined this in regard to FLACS in published literature. The FEMCAT trial (56) confirmed this hypothesis and concluded that FLACS was more expensive and less effective as compared to PCS, with an incremental cost-effectiveness ratio (ICER) of € 10,703.2 saved per patient successfully treated with PCS. While the FACT trial found FLACS to be non-inferior to PCS, FLACS was also not cost-effective (86).

Roberts et al. (87) designed a model that could make FLACS cost-effective only if efficiency increased by 100% or if the patient interface cost was reduced by 70%.

### Learning curve for FLACS

FLACS, similar to any new surgical technique, has a learning curve even for experienced surgeons. Studies have found that, after ~100 cases, the complication rate drops significantly (20, 88). Surgeons familiar with refractive femtosecond lasers may learn FLACS faster (88). Improvements in training techniques and laser software can also contribute to significantly flattening the learning curve (89). However, a survey showed that 82.4% of surgeons felt that 10 cases or less require FLACS to be performed with the same safety as PCS (90).

Although FLACS should also be performed by surgeons who have already mastered PCS, some studies showed that senior residents had similar surgical outcomes between PCS and FLACS (81, 91, 92).

## Discussion and perspectives

Femtosecond laser use does not have to be confined to biological tissue interaction only; they can theoretically alter properties of an already implanted IOL changing its refractive index and, in turn, overall power, toricity, inducing/erasing multifocality, or generating pinhole apertures. Such concepts have only been tested *in vitro* so far (93–95). These techniques might decrease rates of IOL exchange, as those are related to multifocal intolerance and lens power error by 15 and 16%, respectively (96).

Capsulorhexis-fixated IOLs can theoretically benefit from the perfectly round, well-centered, and adequately sized laser capsulotomy (97). Future studies are needed to evaluate whether this will improve ELP and refractive outcomes.

Femtosecond laser technology is likely to evolve as well. Shorter pulse durations could enable higher peak power with less energy per pulse, and a multispot laser treatment could shorten the treatment time. Robotization of the steps of lens aspiration is in its infancy in cataract surgery (98, 99), but this technology could be integrated with FLACS to create fully automated cataract extraction platforms in the future.

## Conclusion

Although FLACS offers precise and reproducible technical performances, in its current state of development, these advantages do not significantly translate into clinical practice. To date, FLACS has not been able to tackle most frequent and burdensome complications of cataract surgery such as residual refractive error [24.9 vs. 22% of eyes with an absolute refractive error >0.5D in FLACS and PCS groups, respectively, in FEMCAT (56)], CME, endothelial cell loss, or vitreous loss. Furthermore, FLACS is a technological adaptation from corneal laser surgery aiming to replace steps of PCS, but the use of phacoemulsification machines is still needed to remove lens pieces and viscoelastic devices. However, although FLACS does not seem to benefit the most typical patients with cataract, it may assist in more challenging cases such as traumatic cataracts, Fuchs's dystrophy, low endothelial cell count, and some pediatric cases. In addition, despite the precise and reproducible technical performances of FSL platforms, FLACS remains in its infancy of development. Indeed, the refinement of lasers, the development of new technologies to replace phacoemulsification machines, or new IOL technologies may help improve clinical outcomes and cost-effectiveness over the conventional PCS.

## Author contributions

MK and CS have conducted literature research and have written the manuscript. Both authors contributed to the article and approved the submitted version.
